# Gastric Anisakiasis Masquerading as Gastroesophageal Reflux Disease

**DOI:** 10.1155/2023/8635340

**Published:** 2023-02-08

**Authors:** Kevin Groudan, Tiago Martins, Ira J. Schmelkin

**Affiliations:** Division of Gastroenterology, University of Massachusetts Medical School-Baystate, Springfield, MA, USA

## Abstract

Anisakiasis of the gastrointestinal tract is caused by the consumption of raw or undercooked seafood infected with *Anisakis* larvae. Penetration of *Anisakis* larvae into the gastrointestinal mucosa leads to severe epigastric pain, nausea, and vomiting, usually within hours of ingestion of the parasite. Suspicion for gastrointestinal Anisakiasis should be raised in patients with a compatible dietary history. Definitive diagnosis can be made by direct visualization of larvae via endoscopic examination. Although symptoms are self-limiting, the removal of larvae by gastroscopy can hasten relief of symptoms. There are a large number of cases of gastric anisakiasis reported from Japan, Korea, and Western Europe, where it is customary to consume raw fish. Cases reported from the United States are less common, and given the nonspecific symptoms of anisakiasis, the diagnosis can be missed. We report a patient who presented with gastroesophageal reflux disease like symptoms that started after ingesting raw fish. He was found by esophagogastroduodenoscopy to have white, filiform worms penetrating into the wall of his stomach, consistent with a diagnosis of gastric anisakiasis.

## 1. Introduction

Anisakiasis is a zoonotic parasitic disease caused by the accidental ingestion of larval nematodes of the genus *Anisakis* [1]. Most cases identified are infected by *Anisakis* simplex, which belongs to the order *Ascaridida*, family *Anisakidae*, and subfamily *Anisakinae* [2]. Fish or cephalopod species can be parasitized by *Anisakis* larvae [2]. Salmon, tuna, squid, codfish, sardines, and anchovies are among the most commonly parasitized species [2]. Humans are an accidental host of the parasite. In humans, *Anisakis* have been found in the mucosa of the stomach and intestine and less commonly in the omentum, liver, pancreas, and lung [3–5]. Clinical manifestations of enteric anisakiasis include colicky abdominal pain, nausea, and vomiting [6]. Enteric anisakiasis should be suspected in patients with gastrointestinal symptoms after ingesting raw fish; a definite diagnosis is made by endoscopic examination [6]. Enteric anisakiasis is usually managed conservatively with analgesic drugs because the larvae die within one week in the human body [6]. Endoscopic removal of larvae from the stomach can hasten relief of symptoms [6]. We report a rare case of gastric anisakiasis in a patient who presented with gastroesophageal reflux disease (GERD) symptoms.

## 2. Case Presentation

A 34-year-old American man with a past medical history of chronic GERD presented with 3 days of postprandial, burning epigastric and throat pain, nausea, and a dry cough concerning for recurrence of his GERD symptoms. He reported taking pantoprazole 40 mg twice daily without improvement in his symptoms. He had an esophagogastroduodenoscopy (EGD) performed by an outside provider four months prior with findings concerning for Barrett's esophagus. He requested a second opinion and a repeat EGD with a biopsy to reevaluate for Barrett's esophagus.

**Figure 1:**
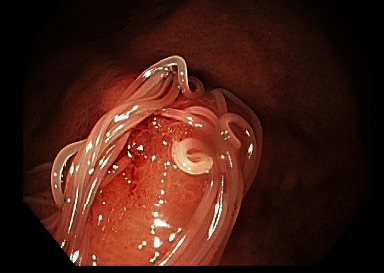
White, filiform worms invading the greater curve of the body.

**Figure 2:**
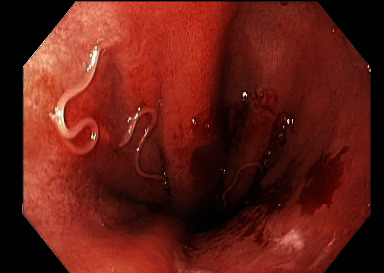
White, filiform worms invading the gastroesophageal junction.

**Figure 3:**
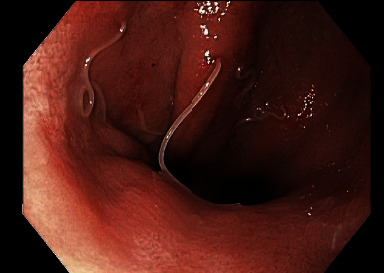
White, filiform worms invading the antrum.

An EGD was scheduled forty days later. At the time of his EGD, he was still symptomatic. His EGD revealed patches of erythema in the stomach associated with white, filiform worms invading into mucosa of the greater curve of the body and the cardia at the gastroesophageal (GE) junction (Figures 1–3). Multiple cold forceps biopsies of the stomach and worms were performed for both pathology and microbiology-parasitic identification. All the worms (approximately a dozen) were extracted using biopsy forceps. Examination of the esophagus also revealed salmon colored mucosa suggestive of Barrett's esophagus. Biopsies of the esophagus were obtained. The patient noted complete resolution of his symptoms immediately after the procedure. Biopsies of the stomach returned showing chronic, active erosive gastritis, and the filiform worms were reported to be morphologically consistent with *Anisakis* nematodes. Biopsies of the esophagus revealed focal intestinal metaplasia consistent with Barrett's esophagus. A follow up EGD performed sixty-nine days after the index endoscopy confirmed complete clearance of the parasite, and the patient reported no recurrence of his symptoms.

In hindsight, the patient reported that for several months leading up his symptoms, he ate raw fish at least once weekly. Specifically, he purchased raw salmon and another raw fish, whose name he does not recall at his local grocery store and consumed both marinated but uncooked. He reported consuming raw fish as recently as a few days before his index endoscopy and stopping raw fish consumption after discovering he was infected with anisakiasis.

## 3. Discussion

The first case of anisakiasis was reported in 1876 by Leuckhart [7]. In the 1960s, the disease became widely recognized when epidemics of anisakiasis occurred in the Netherlands with the increased popularity of lightly salted herring [8]. Since then, many cases have been reported, especially in Japan, where raw fish is routinely consumed [9]. 2,000 to 3,000 annual cases of anisakiasis are reported in Japan alone [10]. Other countries where anisakiasis is commonly reported include Korea, China, Malaysia, Taiwan, Spain, Italy, France, and Germany [11].

Although there are fewer cases of anisakiasis reported in western countries, Americans should remain vigilant. According to the 2005–2010 National Health and Nutrition Examination Surveys, over 80% of Americans reported consumption of seafood in the prior 30 days [12].

Anisakiasis can be misdiagnosed as a peptic ulcer, gastritis, or gastroesophageal reflux disease due to its nonspecific symptoms [1]. Our patient's burning throat and epigastric pain, nausea, and dry cough in the setting of his known GERD was suspicious for recurrence of his GERD symptoms; however, given that the onset of his symptoms was associated with his new diet rich in raw fish, and his symptoms resolved after his anisakiasis were removed endoscopically, his presentation was much more likely due to anisakiasis. Symptoms of anisakiasis include acute epigastric pain, which can occur a few hours after ingestion of the parasite [1]. Other reported symptoms have included nausea, vomiting, and low-grade fever, which is reported to occur within 5 days after consumption of the parasite [13]. There are rare cases of patients presenting with hematemesis as a result of gastric ulceration, and some cases are asymptomatic and incidentally diagnosed [13].

The pathogenesis of anisakiasis includes hypersensitivity to the larva or its secretions and mucosal injury as a result of larvae penetration [11]. When *Anisakis* larva die after mucosal penetration, eosinophil infiltration and connective tissue proliferation occur around the larval body forming an eosinophilic granuloma [14]. There are some cases of anisakiasis granulomas mistaken as a submucosal tumor [2]. The survival time of *Anisakis* in humans is short, as they are usually expelled or destroyed within a few days to a week [2].

Gastric anisakiasis is diagnosed by direct visualization of larvae by EGD [1]. The most common endoscopic finding is gastric mucosal edema surrounding the zone of penetration [15]. Our patient's endoscopic examination revealed mucosal erythema surrounding the larvae. *Anisakis* appear to have a predilection for invading the greater curvature of the stomach [16]. Our patient's larvae were present in the greater curvature of the stomach and cardia at the gastroesophageal junction. Laboratory studies showing an elevated white blood cell count with eosinophilia can assist with the diagnosis of anisakiasis; however, a case series in Korea showed that eosinophilia is not commonly seen in infected patients [17]. Our patient did not have laboratory studies performed at the time of the infection. Abdominal computed tomography can be useful for ruling out other causes of abdominal pain [13]. Submucosal edema of the gastric wall as well as attenuation of adjacent fat can be seen in patients with gastric anisakiasis [18].

Endoscopic extraction is the mainstay of treatment for anisakiasis [13]. Extraction can be performed using conventional forceps, which were used for our patient [13]. As part of the parasite can be embedded in the gastric mucosa, it is important to ensure complete extraction by grabbing the larva as close to the wall as possible [13]. If not completely removed, larvae can continue to cause inflammation and gastrointestinal symptoms [13]. A thorough examination of the entire stomach is integral, as larvae can infect multiple sites [19]. Endoscopic removal of larvae typically results in prompt resolution of symptoms [13]. In cases managed supportively with analgesic medications, symptoms usually resolve within a week after the larvae are destroyed or excreted [13]. There are some reports of using albendazole 400 mg orally twice a day for 6 to 21 days to treat anisakiasis however data is limited [20].

## 4. Conclusion

Anisakiasis is a rare parasitic disease caused by the accidental ingestion of *Anisakis* larvae from raw or undercooked seafood. A definitive diagnosis is made by endoscopic visualization and endoscopic extraction is curative. Clinical manifestations of gastric anisakiasis are nonspecific and include abdominal pain, nausea, vomiting, and fever, making the diagnosis challenging without endoscopic evaluation. In our case, gastric anisakiasis masqueraded as gastroesophageal reflux disease due to our patient's history of gastroesophageal reflux disease and symptoms of burning throat and epigastric pain, nausea, and a dry cough. Clinicians should consider anisakiasis in the differential diagnosis of patients presenting with gastrointestinal symptoms and a recent history of raw or undercooked fish consumption.

## Data Availability

The data described in this article are available on PubMed.
